# Patterns and Determinants of Imported Malaria near the Argentina–Bolivia Border, 1977–2009

**DOI:** 10.3390/pathogens14060537

**Published:** 2025-05-28

**Authors:** Erica Berlin, Roberto D. Coello-Peralta, Pedro Cedeño-Reyes, Elsa M. Valle-Mieles, Paul L. Duque, Mario O. Zaidenberg, Horacio Madariaga, Juan C. Navarro, María J. Dantur-Juri, Marcia C. Castro

**Affiliations:** 1 Department of Global Health and Population, Harvard T. H. Chan School of Public Health, Boston, MA 02115, USA; erb244@mail.harvard.edu; 2 Department of Microbiology, Faculty of Veterinary Medicine and Zootechnics, Universidad de Guayaquil, Chile y Av. Olmedo, Guayaquil 090511, Ecuador; roberto.coellope@ug.edu.ec (R.D.C.-P.); pedro.cedenor@ug.edu.ec (P.C.-R.); elsa.vallem@ug.edu.ec (E.M.V.-M.); 3 Unidad Ejecutora Lillo (CONICET-Fundación Miguel Lillo), San Miguel de Tucumán, Tucumán 4000, Argentina; polduquebiologo@gmail.com; 4 Coordinación Nacional de Control de Vectores, Ministerio de Salud de la Nación, Salta 4400, Argentina; mozainderberg@gmail.com; 5 Instituto de Estudios Geográficos “Guillermo Rohmeder”, Facultad de Filosofía y Letras, Universidad Nacional de Tucumán, San Miguel de Tucumán, Tucumán 4000, Argentina; madariagah@gmail.com; 6 Research Group of Emerging and Neglected Diseases, Ecoepidemiology and Biodiversity, Health Sciences Faculty, Universidad Internacional SEK (UISEK), Quito 170121, Ecuador; juancarlos.navarro@uisek.edu.ec; 7 Fundación Miguel Lillo, Instituto de Genética y Microbiología, San Miguel de Tucumán, Tucumán 4000, Argentina

**Keywords:** cross-border malaria, epidemiological surveillance, human migration, malaria

## Abstract

In the Americas, the number of confirmed malaria cases decreased by 65.4% between 2000 and 2023, and malaria elimination is now in sight for many countries. Argentina is currently free of autochthonous malaria transmission. Until 2011, cases of malaria were concentrated in Northwestern Argentina, near the border with Bolivia, a country that continues to have malaria transmission. The Orán department (Salta province, Northwestern Argentina) had particularly high transmission near a main road that is a pathway for migration from Bolivia. The purpose of this study was to identify which factors best explain the extent and timing of changes in the proportion of malaria cases in this area that were locally transmitted versus acquired in another country. Combining information from routinely collected case investigations, epidemiological surveillance data, and satellite imagery, we used a logistic model and a multi-level model of change to identify how demographic and place-level variables influence the proportion of malaria cases that were imported over time. The findings showed that the proportion of cases that were imported varied significantly over time, with a clear trend from predominantly autochthonous cases at the beginning of the study in 1977 (94.52%) to a majority of imported cases in 1992 (53.33%), a pattern that continued and intensified, reaching 76% imported cases by the end of the series in 2009. Nationality and place of work were key demographic factors influencing this shift. In particular, there was a change in transmission patterns after a cross-border intervention was launched in 1996. As Argentina has obtained certification of malaria elimination, these results may inform focal strategies for preventing re-introduction.

## 1. Introduction

In 2023, malaria, an acute febrile illness, led to 263 million cases globally. This represents an increase of 11 million cases compared with 2022, and over 597,000 deaths [1]. In 2020, malaria deaths increased by 12% compared with 2019. This increase is probably due to disruptions in the surveillance and control activities of the disease during the COVID-19 pandemic [2]. In the Americas, the number of confirmed malaria cases decreased by 65.4% between 2000 and 2023 [1]. In Argentina, there have been zero locally acquired cases since 2011; the country obtained certification of malaria elimination in 2019, and according to the latest report from the World Health Organization, it maintains this status [1].

Malaria elimination first became a priority in Argentina in 1876 when farming was expanded [3]. Later, in the 1900s, several control methods to kill larvae and adult anophelines in malaria-endemic areas were implemented [4,5]. Overall, these interventions failed because they were not tied to the local vector behavior. Initially, malaria research in Argentina was heavily influenced by European malariologists. Around the 1900s, many Argentine researchers began studying disease dynamics in the Argentine context. They discovered that the most common malaria vector was *Anopheles pseudopunctipennis,* a species whose larvae were mainly found in streams with algae of the *Spirogyra* genus and little herbaceous vegetation [6,7]. This differed greatly from previous assumptions (largely based on research in European countries) that most common larval habitats were swamps and marshes [5]. During the 1930s and 1940s, the malaria eradication program was focused on finding and treating only *An. pseudopunctipennis* larval habitats and killing adult mosquitoes [8].

By the early 1950s, there were a few hundred cases per year, which were mostly concentrated in Northwestern Argentina. Yet as agriculture, ranching, logging, and oil exploration expanded on the northern border with Bolivia, the number of malaria cases around the cities of Orán and Tartagal surpassed 2500 in 1959. Bolivia had been much less successful in controlling malaria, and thus, this shared border region had a unique set of public health challenges. During the 1960s, there was a slow but steady decline in case numbers across Argentina, except for Salta and Jujuy provinces [5]. New cases were related to human migration flows along the Argentina–Bolivian border, particularly from the Tarija department (Bolivia) to the Orán department (Salta Province, Argentina), along National Route 50 (the major highway in the Orán department). National Route 50 is 71 km long and crosses National Route 34 at Pichanal city, where the international bridge on the Bermejo River, a natural border between Bolivia and Argentina, can be found. This route is considered an important migration path between Bolivia and Argentina.

Human movement into the Oran department was primarily associated with seasonal work on farms, where individuals harvested crops such as sugarcane, fruit, and vegetables. Migrant laborers generally worked in a specific locality for a defined period, returning to their home country at the end of the harvest season. The laborers were paid daily and rarely moved between localities during the harvesting. Migrants who crossed the official border are likely to be investigated through routine epidemiological surveillance [3]. There are also informal crossing points along this border where mostly rural workers enter and settle illegally without having the required documents. Those who crossed through informal routes are difficult to track, and thus, underreporting of imported cases is expected [3]. Their stay in the country becomes difficult; in addition, there is a risk of migrating during the disease incubation period and then, when the first symptoms appear, resorting to traditional healers or pharmacies to seek medication for their fever. Only when complications appear or when the symptoms do not disappear with medication do these people seek services or go to health centers for the necessary attention. In the 1990s, the number of malaria cases in the province of Salta surpassed those in other provinces with endemic malaria across Argentina. In 1996, cases along the Argentina–Bolivia border increased, reaching a peak of over 20,000 cases, with 2076 in Argentina and 22,331 in Tarija, Bolivia. This surge was likely exacerbated by migration and climate fluctuations [9]. In the same year, health officials in both Argentina and Bolivia launched an intervention program called ArBol (Argentina–Bolivia), which included surveillance, control (residual house spraying and detection of patients with fever), and treatment [3].

The program mainly focused on the “Bermejo Triangle”, an area bounded by the Bermejo and Tarija rivers, which connects to the city of Orán. It was intended to have spillover effects on the surrounding areas. The malaria cases progressively declined, and the program ended in 2001 [5]. Since 2000, 90% of Argentina has been considered malaria-free, with only reports of imported cases, mainly from Bolivia [3,10]. In 2008, the endemic area, where the last cases of malaria were recorded, was stratified into two risk areas or strata. The first risk area, including the Orán and San Martin departments within Salta province, had about 4000 homes, mainly in rural areas that are difficult to access, and had the highest malaria risk in the country. The second area included the El Carmen, Palpalá, General Belgrano, San Pedro, and Ledesma departments (Jujuy province), the Anta department (Salta province), and Puerto Iguazú municipality (Misiones province, but no locally transmitted cases). The San Martín department has had the highest numbers of locally transmitted and imported cases since 2007. On the other hand, the department of Orán has had zero cases of local transmission during the same period and no cases at all since 2011. Since 2009, the majority of malaria cases in Salta province have been classified as imported [3].

In Bolivia, a 70% reduction in malaria cases was recorded between 1998 and 2014 [11]. However, in 2023, the number of cases increased by between 24% and 63% compared to 2015 [1]. While the northern region of the country is considered the highest-risk area for malaria transmission, the department of Tarija, which borders the Oran department, continues to hold a low-risk status, with zero to three isolated cases reported in recent years [1,11].

Both migration (long-term relocation) and human mobility (broader movement, including commuting and temporary travel) can affect malaria transmission [5], posing a risk for (re-)introduction as infected individuals migrate into receptive areas [12,13,14]. Focusing on Latin America, massive population movement into the Sucre state, Venezuela, for gold mining was also associated with a major increase in imported malaria cases [15]. In addition, movement of workers from endemic areas to non-endemic areas has been shown to lead to reinfections of local mosquito populations, which has occurred in areas of Brazil outside of the Amazon region, where the majority of transmission takes place [16]. More recently, migration of Brazilians into Suriname and French Guiana to work in gold mining has been associated with increases in malaria around mining sites in the two countries [17,18]. In addition, commuting to work at hydropower dam regions in Porto Velho municipality, Rondônia, Brazil, as well as the return of urban residents to surrounding rural areas, has been linked with malaria transmission [19].

Past studies using monthly registry data have effectively analyzed historical trends. These analyses indicate that migration presents a significant challenge for malaria control and elimination [14,15,20,21,22]. Other studies have shown that predictions based on case data can be improved by incorporating factors related to breeding sites and proximity to human populations, which serve as a source of blood meals, that can be ascertained through spatial analysis [6,9,21,22]. While most studies have traditionally focused on either geographical differences or temporal changes in malaria rates, a multi-level model offers a more comprehensive approach by simultaneously accounting for both types of variation. This capability makes it particularly useful for analyzing changes in malaria consultation rates [23], as well as data from malaria case investigations [24] and routine surveillance data, where both spatial and temporal factors can be influential [1].

In Argentina, the majority of recent research on malaria has taken a historical or political economy lens, and focused on the DDT era and prior decades [2,4,5,7,8]. In addition, studies have focused on the influence of the Normalized Difference Vegetation Index (NDVI), Land Surface Temperature (LST), and climatic variables on *Anopheles* mosquitoes’ presence and abundance over time in malarious areas [20,25,26]. There is a gap in the literature regarding factors associated with past decreases in locally transmitted malaria cases in Argentina. In particular, no research has been conducted on how demographic differences between imported and locally transmitted cases contributed to changes in malaria transmission over time. Therefore, this study aims to fill this research gap.

The goal is to identify individual- and place-level factors that best explain the extent and timing of changes in the proportion of imported malaria cases in the Orán department near National Route 50, but also to assess if and how the proportion of malaria cases that were imported differs before and after the cross-border ArBol intervention. Furthermore, we have three hypotheses. First, workers who are day laborers (with a salary provided per day of field work) or have professions that involve frequent cross-border travel are at the highest risk of being infected and thus contribute to the importation of malaria cases. Second, people who live closer to the border and near manually farmed crops will be more likely to be imported cases, but proximity to vectors will have less effect on one’s chances of being an imported case. Third, we hypothesize that the proportion of cases imported will be higher after 1996, and the relative proportion of cases that are locally transmitted will decrease along with the absolute number of cases.

## 2. Materials and Methods

### 2.1. Study Area

**Figure 1:**
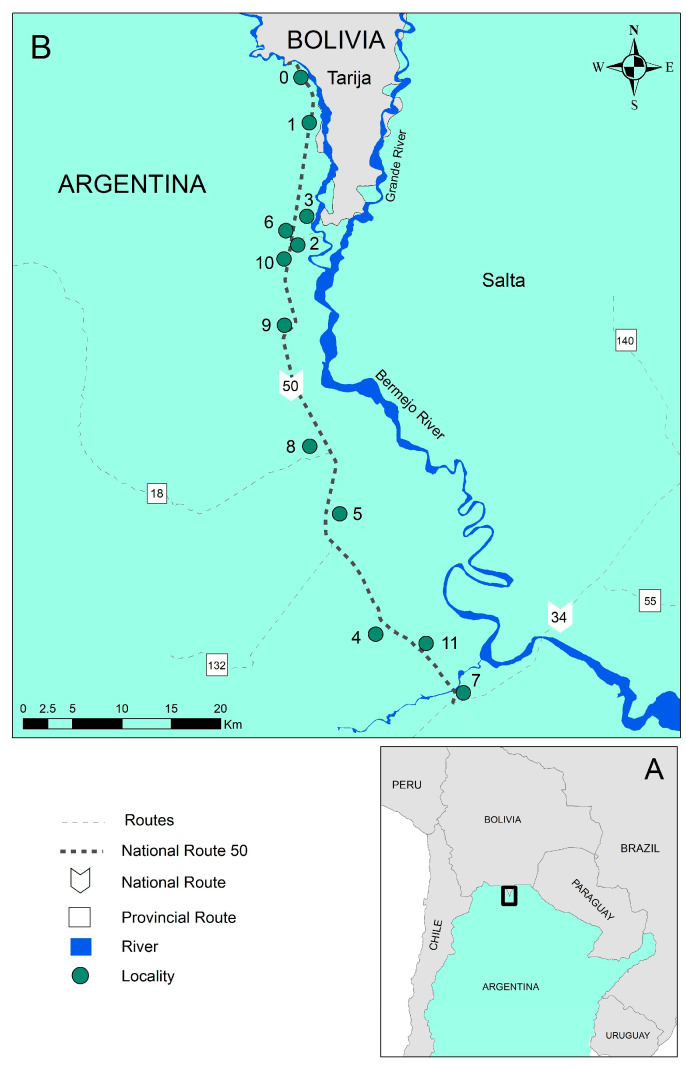
(**A**) Study area located near the Argentina-Bolivia border. (**B**) Localities near Route 50, Orán department, Salta province, Northwestern Argentina (0: Aguas Blancas; 1: El Cebilar; 2: El Pelícano; 3: Finca La Esperanza; 4: Hipólito Irigoyen; 5: San Ramón de la Nueva Orán; 6: Peña Colorada; 7: Pichanal; 8: Río Blanco; 9: Río Pescado Puente; 10: Solazutti; and 11: Tabacal).

The study area includes 12 localities near National Route 50 in the Orán department in Salta province (Figure 1): Aguas Blancas (urban locality; 22°43′82″ S, 64°21′54″ W), El Cebilar (rural; 22°43′57.68″ S, 64°21′8.63″ W), El Pelícano (rural; 24°3′27.80″ S, 63°43′1.08″ W), Finca La Esperanza (rural; 23°7′ 47″ S, 64°19′18″ W), Hipólito Irigoyen (urban–rural; 23°14′45″ S, 64°16′26″ W), San Ramón de la Nueva Orán/SRN Orán (urban; 23°8′13.4″ S, 64°19′27.4″ W), Peña Colorada (rural; 23°45′0″ S, 63°55′0″ W), Pichanal (rural; 23°18′48.2″ S, 64°13′11.1″ W), Río Blanco (rural; 23°3′47″ S, 64°16′31″ W), Río Pescado (rural; 22°56′50.74″ S, 64°21′56.61″ W), Solazutti (rural; 22°54′0″ S, 64°19′59.88″ W), and Tabacal (rural; 23°15′0.1″ S, 64°14′40.2″ W). The localities range in size, including larger towns and smaller farming communities. The town of Orán has the largest population, with 66,915 residents according to the 2001 census, and a growth rate of 0.03% since the previous census. The smallest locality, El Cebilar, had merely 377 residents in the same year [14]. The main uses of land in the area include industrial sugar farming, rice farming, fruit tree and small vegetable cultivation, and logging [25]. The distance between the northernmost locality in the Óran department (Aguas Blancas) and the southernmost locality (Pichanal) is 72.9 km. The average altitude is around 307 m above sea level (masl).

The study area is located in the Sierra Baja of Orán, and thus is situated in the geological province of the Sub-Andean Sierras. Additionally, the area is characterized by fast-flowing rivers [27]. This route is considered an important migration path between Bolivia and Argentina, and three seasons can be recognized along it: a warm dry season corresponding to the spring (September–December), a hot wet season corresponding to the summer (January–April), and a warm–cold and humid season corresponding to the autumn and winter (May–August) [27]. In 1996, a year with high malaria incidence, the mean temperature and precipitation in the Aguas Blancas locality (Orán department) were 29.5 °C and 111.4 mm, respectively. In 2003, a year with a low incidence, the mean temperature and precipitation were 29.1 °C and 148.58 mm, respectively. Meanwhile, in 1996, the mean temperature in the Orán city locality (Orán department) was 28.1 °C, and precipitation totaled 88 mm. In 2003, the mean temperature and precipitation were 31.4 °C and 96.8 mm. The malaria transmission season historically occurred in the area from December to March [20].

In the study area, the primary vector was *Anopheles pseudopunctipennis,* with secondary vectors such as *An. argyritarsis*, *An. strodei*, and *An. evansae* [25,26]. The main malaria parasites historically present in the region include *Plasmodium vivax*, *P. falciparum*, and *P. malariae. Plasmodium vivax* accounted for 99% of cases since the previous malaria certification [5,8,20]. Standard interventions implemented in the region, particularly since the national eradication program was initiated in 1959, have included identifying carriers, preventing and treating residual or imported infections, and indoor residual spraying. Surveillance strategies involved active and passive case detection, epidemiological investigation, treatment with standardized medications, and targeted insecticide spraying of affected and nearby households [3].

### 2.2. Data Sources

Due to the retrospective nature of the data, no variables were manipulated, and individuals were not tracked in real time. The Ministry of Health of Argentina provided data sheets and samples that had been previously approved for collection by the Ethical Committee. This was carried out in accordance with the methodology described in the Protocol of the Manual de Normas y Procedimientos de Vigilancia y Control de Enfermedades de Notificación Obligatoria, Ministerio de Salud de Argentina, which required documented verbal agreement from all participants involved in this study.

Data on monthly malaria cases recorded in the study area from 1977 to 2009 were extracted from epidemiological surveillance sheets that were filled out when each case was investigated. The study population was defined to include all individuals diagnosed with malaria using thick and thin smears taken as a health routine or during a case investigation. Moreover, each case was also interviewed about their travel history and proximity to travelers. The data are estimated to have been accurate, as answering questions would not lead to a change in their migration status. Even if a suspected patient was detected, samples were taken from cohabitants and neighbors [3,23]. The sheets were included in this study if their registered address was within one of the 12 study localities.

Based on the above information, we created two outcome variables: (i) the proportion of imported malaria cases, and (ii) a binary variable indicating whether a malaria case was imported. We restricted the sample to cases that listed their home address as one of 12 localities near National Route 50.

For each case recorded, we also obtained information on gender, nationality, age, type of occupation, country of work, and locality of infection. Age was categorized as <5, 5–9, 10–19, 20–29, 30–39, and ≥40 in order to capture differences between high-risk young children, older children, and younger adults. The type of occupation was considered in the following categories: homemaker, entrepreneur, domestic worker, day laborer, not working, or other for those missing an entry for occupation, to observe the higher risk of locally transmitted malaria [3]. Locality of infection was assigned by case investigators based on the patient’s travel history.

Land use information was obtained from classifying a Landsat 7 satellite image taken in February 2003 from the United States Geological Survey (USGS) Earth Explorer (earthexplorer.usgs.gov, accessed on 22 May 2025). Based on that information, distance-based measures were calculated for each one of the 12 localities: distance to a larval habitat proxy, distance to forested areas, distance to manually farmed crops, distance to the border, and distance to National Route 50. The variables were assumed to remain constant over time, as they did between an image from 1995 and the one used. Although a considerable timeframe separates the images, the selected land use variables were chosen due to their relative stability over time. The review of historical imagery up to 2003 suggested that, while some land use changes may have occurred, the spatial configuration of key features, such as forested areas, major agricultural zones (represented by manually farmed crops), and the primary transportation network (National Route 50), remained relatively consistent. The agreement between the images reinforces this assumption and was considered the best possible approximation.

As no exact coordinates of larval habitats were available, their larvae tend to concentrate in the low-lying ends of rivers and streams, and *An. pseudopunctipennis* mosquitoes typically grow in sun-exposed clean freshwater—particularly bodies of water that contain filamentous algae—these parts of bodies of water were used as a proxy for larval habitats [5]. Distance to larval habitats was categorized as less than vs. greater than 5 km because *An. pseudopunctipennis* tends to have a flight range of 4–6 km [5]. Second, distance to the edges of forested areas was analyzed because the *Anopheles* species in the study area were found to have the highest abundance concentrated near the edges of forested areas compared with forest and peridomestic areas [3]. Again, based on the flight range, the cutoff for distance to the edge of forested areas was also 5 km. Third, the distance to manually farmed crops was selected because day laborers tend to be migrants from Bolivia and are often used to farm medium-sized plots of land. These types of plots were identified, and whether a locality was within 5 km of such a plot was measured as a proxy for migration. The fourth variable was distance to the border, and the cutoff was 10 km. A thorough literature search revealed that there has been limited study on the relationship between malaria and borders. The distance to the border cutoff was 10 km because the one study that examined this association found that malaria incidence in China was highest within 10 km of an international border [28]. The last variable, distance to National Route 50, was categorized as more or less than 1 km. All of the localities were close to National Route 50 by design, and thus, a small cutoff was most appropriate.

### 2.3. Statistical Methods

For the purpose of exploratory data analysis, we conducted chi-square tests to measure the association between the response variable and each individual-level variable, locality, and locality level variable, as well as the differences between autochthonous and imported cases. In addition, correlations were run between the response variable and each locality level variable.

In order to assess if and how the proportion of malaria cases that were imported differs before and after the cross-border ArBol intervention launched in 1996, we fitted a logistic model using the binary outcome variable indicating if the case was imported. The unit of analysis of this model is the locality, and the covariates used were a binary variable indicating if the cases were observed before or after 1996, as well as distance to larval habitats, forested areas, manually farmed crops, the border, and National Route 50.

In order to identify individual- and place-level factors that best explain the extent and timing of changes in the proportion of imported malaria cases, we specified a two-level multi-level model of change (months nested within localities). The response variable is the proportion of imported cases by month in each locality. Individual-level variables were aggregated to the locality. Three models were run. First, Model A, an unconditional means model that assumed that each locality had the same trajectory over time. Second, Model B is an unconditional growth model that incorporates the time component to assess inter-locality variation across months. Third, Model C, a full model with the following covariates as time-varying predictors: gender, age groups, occupation, locality of infection, and locality of work. In addition, to account for ArBol intervention, we added the binary variable indicating whether the case was diagnosed before or after January 1996. Model goodness of fit was assessed with the Akaike Information Criterion (AIC). All models were run in Stata 14 (StataCorp LLC, College Station, TX, USA) [29].

## 3. Results

### 3.1. Malaria Transmission for the Years 1977 to 2009

Between 1977 and 2009, 1775 people were diagnosed with malaria who had addresses in 11 of the 12 localities near Route 50 (Table 1). One locality, Solazutti, had zero recorded cases throughout the study period. Probably, being a smaller locality, it did not require a large workforce, and it is possible that its inhabitants did not present the disease, did not manifest malaria symptoms, or even if they did, did not seek medical attention. Homemakers and those not working tend to be at higher risk of locally transmitted malaria. Entrepreneurs, domestic workers, and day laborers are more likely to be migrants and/or to frequently travel across the border for work or to visit relatives.

All of the cases were diagnosed with *Plasmodium vivax*. The percentage of autochthonous and imported cases differed significantly between genders, nationalities, previous infection status, age groups, occupations, countries of work, origins of infection, and home localities. The most important percentages shown by categories in malaria cases were male (73%), ≥40 age (28%), and day laborer occupation (44.28%). Moreover, in approximately 28% of cases listed, the place of work locality was in Bolivia. Detailed data and significant values are presented in Table 1. The entrepreneur category was also important within import cases (84%), including merchants who frequently travel across the border, and had a higher percentage of imported cases. The percentage of cases that were autochthonous and imported also differed significantly by distance from forested areas, distance to manually farmed crops, distance to National Route 50, and distance to the Bolivian border (Table 1).

**Figure 2:**
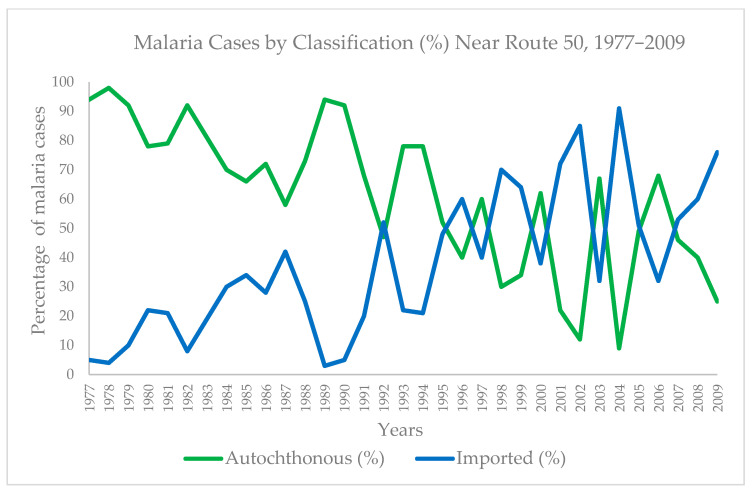
Percentage of malaria cases autochthonous and imported by year (1977–2009).

Overall, 71.27% of cases were transmitted locally, while 28.73% were considered imported from another country. The proportion of locally transmitted to imported cases varied by year. However, until the 1990s, the percentage of locally transmitted cases was consistently higher than that of imported cases. At the start of the study period in 1977, 94.52% of cases were autochthonous and 5.48% were imported. In 1992, imported cases surpassed autochthonous cases for the first time (46.67% autochthonous vs. 53.33% imported). By the end of the series in 2009, imported cases (76%) surpassed autochthonous cases (24%) (Table 1 and Figure 2).

### 3.2. Place-Level Determinants of Imported Malaria Cases

Distance to forested areas, larval habitats, manually farmed crops, and the border with Bolivia had significant effects on malaria importation into the localities. The effects of time-period and locality-varying variables on the odds of being an imported case are presented in Table 2. The distance from National Route 50 was not significant. After adjusting for the locality-level variables, those diagnosed after 1996 had 5.46 times the odds of being an imported case compared with those diagnosed before 1996 (Table 2).

### 3.3. Patterns of Malaria Importation Across Localities and Time

The proportion of cases that were imported was close to zero at baseline but increased over time, particularly after the ArBol intervention began. The results of fitting three multi-level models of change to assess changes over time and across localities in the proportion of cases that were imported are shown in Table 3. Model A indicated that the mean proportion of imported cases across localities was 32.25%. There was statistically significant evidence that the average proportion of imported cases varies across time. However, the level 2 variance was 0.0117, and there was no statistically significant evidence that localities differ from each other in the proportion of cases imported. A total of 7.17% of the total variation in the proportion of imported cases was attributable to differences among localities. As neither of the variances for initial status and rate of change in level 2 was significant, it can be said that they may not be different from zero. Model B indicated that at baseline, the average proportion of imported cases was not different from zero. Yet, for each month, there was a statistically significant increase of 0.0018 in the proportion of imported cases on average.

Covariates were introduced into the fixed effects portion of the model using a stepwise forward approach. Adding a discontinuity term before the ArBol intervention began implementation vs. after lowered the AIC. This suggests that the rate of change in the proportion of cases that are imported does change after the intervention. In the final model, Model C, the overall average proportion of imported cases at baseline was 0.0069, and there was insufficient evidence to suggest that it differed from zero. There was statistically significant evidence that the rate of change decreases by 0.0006 each month for those over 40 years of age compared with the reference group. Yet no significant effects were found for gender, age, or occupation.

The difference between the proportion of imported cases before and after the intervention was 0.2008. The difference between the rate of change in the proportion of imported cases in the two time periods was 0.0016, indicating that the proportion changed at a slightly higher rate after 1996. The within-locality variance was lowest in Model C, at 0.1057; however, it was still statistically significant, indicating that there was further unexplained within-locality variance. A further 33.40% of within-locality variance was explained by adding the covariates in this model. The level 2 variance at baseline and for rate of change was still not statistically significant, indicating that there may not be variation across localities at baseline or in terms of their rate of change. However, it is also possible that there was not enough power to detect differences between localities. In this model, 97.78% of the between-locality variance was explained by time.

## 4. Discussion

This study assessed the changes in the proportion of imported and autochthonous cases in the department of Orán, Argentina, between 1997 and 2009, particularly before and after the cross-border ArBol intervention. Autochthonous malaria refers to cases acquired locally through mosquito transmission within the study area, encompassing both indigenous cases (transmission in an area of regular occurrence) and introduced cases (transmission from an imported case in an area where malaria is not regular). Imported malaria, in contrast, denotes cases where the infection was acquired outside the study area; in our context, this indicates infections acquired across the border in Bolivia. Our results show that the extent of changes in the proportion of cases that were imported was partly explained by how far one lives from larval habitats and forested areas, as imported cases were more likely to be located farther away from these areas. Imported cases had 81% lower odds of living within 5 km of larval habitats and forested areas compared with autochthonous cases. Considering these definitions, this finding suggests that individuals residing farther from potential breeding sites and areas of local transmission risk were less likely to acquire malaria locally (autochthonous infection). However, their greater distance from these local transmission foci did not protect them from acquiring the infection elsewhere, likely across the border, thus presenting as an imported case upon detection in Argentina. In addition, those who lived within 10 km of the border had 55% higher odds of being an imported case, which supports our hypothesis that those who lived closer to the border were more likely to have been infected in Bolivia. This finding is likely due to the proximity to the Bolivian border region, specifically the Tarija department, which is known to be endemic for malaria transmission.

While detailed data on the historical malaria situation on the Bolivian side and the precise dynamics of migration and mobility in this frontier were limited due to information-sharing constraints and potential reporting biases in our data, the established endemicity across the border strongly suggested a significant source of imported cases. The epidemiological context of this Argentina–Bolivia border region, as noted by previous research, highlighted the strong influence of migration flows and the interconnectedness of vector-borne disease dynamics, with malaria epidemics in Argentina often linked to similar situations in Bolivia. The overlapping social, cultural, and epidemiological spheres of border towns further underscore the likelihood of cross-border infection. Overall, the percentage of cases involving day laborers exceeded that of cases in other professions. Their general dynamic usually included the transfer from the border to the cultivation area, their location in rural areas, potentially near mosquito breeding sites, and limited access to preventive measures. This finding is consistent with Cuellar’s observation that the majority of cases in the city of Orán between 1986 and 2000 involved day laborers [30]. Yet the fact that imported cases tend to live farther from these farms seems to contradict the hypothesis that day laborers were more likely to be migrants at risk of importing malaria. Controlling for place-level variables, the logistic model indicated that the odds of being an imported case were over 5.46 times higher after 1996 compared with before 1996. This result suggests that local transmission was significantly lower after the cross-border intervention began implementation.

The multi-level models provided further evidence that the majority of the variation in the proportion of malaria cases that were imported was due to variations within localities over time rather than across localities. The unconditional mean model (Model A) provided evidence that the proportion of imported cases varies across time, and the unconditional growth model (Model B) indicated that 61.54% of the between-locality variation and 18.2% of the within-locality variation was explained by time. Models B and C indicated locally transmitted cases were far bigger drivers of transmission than imported ones at the beginning of the study period. Yet, both models indicated a statistically significant change over time. Specifically, Model C found a 0.16% increase in the proportion of cases that were imported on average per month. This provides convincing evidence that over time, the amount of local transmission decreased and the proportion of imported cases increased. This finding is consistent with Zaidenberg’s [3] observation that, since 2008, approximately 50–70% of cases in Argentina have been considered imported, and the absolute number of imported cases has been declining.

Furthermore, adding a dichotomous term representing before and after 1996 significantly improved the multi-level model of change from a model of demographic variables alone. This indicated that the rate of change truly changed in an important way at this point. This was consistent with Zaidenberg’s finding that there was an important increase in cases in the early 1990s and that the outbreak reached its peak on both sides of the border in 1996 [3]. The proportion of cases that were imported was 20.08% higher after 1996, and the estimated difference between the rate of change before and after the intervention was 0.0016. These results suggest that the cross-border intervention may have decreased local transmission and facilitated elimination. These results support Carter’s finding that the ArBol program coincided with a reduction in overall malaria incidence on both the Argentinean and Bolivian sides of the border by 2001, as well as a report that stated that overall prevalence went down by 75% within two years of the intervention [10,30].

The ArBol program was a binational effort that highlighted the different epidemiological situations and control capacities of both countries [3]. While our results indicate a significant decrease in locally transmitted malaria cases following the implementation of ArBol, the persistence of imported cases suggested that the intervention’s impact was more complex. Bolivia historically faced more complex challenges in controlling malaria [1,2], and this difference likely influenced the overall effectiveness in the border region, creating a continuous risk of malaria importation. Future research and interventions should consider an integrated, binational approach to address cross-border malaria transmission dynamics and the effectiveness of its control. Even after achieving malaria elimination, the experience in Sri Lanka underscores the persistent risk of re-introduction from imported cases, particularly in regions with significant cross-border movement. This highlights that the success of interventions such as ArBol in reducing local transmission does not eliminate the requirement for continued vigilance against imported malaria [31].

The chi-square tests indicated that overall, the percentages of cases that were autochthonous and imported significantly differed by sex, age category, job category, nationality, infection history, place of work, and place of infection. Cuellar, in their studio conducted from 1986 to 2005 in Orán, also found that overall, cases were more likely to be male, aged 15–30, and day laborers [30]. In addition, Dantur Juri et al. [20] found that in Aguas Blancas from 1994 to 2004, overall malaria prevalence was highest in people under 20 years of age and young people involved in commercial activities. Yet in Model C, controlling for other variables, there was insufficient evidence that the proportion of imported cases differed across genders, age categories, or job categories. Among those over 40 years old, the rate of change was significantly different from that of the reference group, suggesting that individuals in this age group had a slightly lower rate of change over time compared with those under five. However, there was insufficient evidence to suggest that the rate of change in proportion of imported cases over time differs by gender, age category, or job category. Nevertheless, these variables played an important role in explaining a larger proportion of the variance within localities. The within-locality variance was lowest in Model C, and a further 34.59% of that variance was explained by this model. However, there is still a statistically significant amount of unexplained within-locality variance, which may be due to other demographic aspects that were not captured by the case investigation forms.

This study examined an area in Argentina that has a history of exceptional malaria control efforts and looked at the unique place-level factors of the area. However, it is possible that the general lessons from this study could be cautiously applied to other parts of the Orán department or San Martín department and possibly border areas of other countries in the region, working towards elimination. One potential limitation to this study was that while larval habitats and distance from the center of locality to National Route 50 and border tend to remain stable over time, areas that were used for farming and forested areas tend to change over time due to changes in land use. While preliminary analysis indicated that these last two variables remained constant between the two time points near the end of the study period, the assumption that they remained constant throughout the entire study period may have biased the results. Cuellar found that the Normalized Difference Vegetation Index (NDVI), a measure of the photosynthetic activity of plants, decreased by 2% from 1996 to 1998 alone, and there are no prior studies on land use [30]. Another potential limitation is that this study only included people who had addresses in one of the 12 localities near National Route 50. Since the area near National Route 50 does experience frequent population movement, there are others who were diagnosed with malaria while temporarily working in the area or visiting relatives who were excluded. Since these people did not consider their address to be one of the 12 localities, the ability to capture their contribution to malaria transmission was limited. This limitation is particularly relevant when considering the risk of re-introduction. As seen in Sri Lanka, even after achieving malaria elimination, mobile populations, including temporary workers, can be a key source of imported malaria, potentially leading to local transmission if receptive conditions exist [31]. Therefore, understanding the dynamics of malaria in these mobile populations, even if not permanent residents, is crucial for preventing re-establishment.

The ongoing risk of malaria re-introduction in areas with a history of autochthonous transmission necessitates sustained and robust surveillance and response systems. Furthermore, the experience in Sri Lanka demonstrates the critical importance of continuous monitoring, including active case finding among migrant worker groups and entomological surveillance, even after achieving malaria-free status.

## 5. Conclusions

This study demonstrated that the proportion of malaria cases that were imported in a part of the department of Orán, Argentina, near the border with Bolivia, increased from 1977 to 2009. The rate at which this proportion changed was higher after the ArBol cross-border intervention was launched in 1996, suggesting that inter-country cooperation can play an essential role in reducing malaria transmission and approaching elimination, primarily through the effective reduction in local transmission in the country seeking elimination. This study also showed that individuals who migrated and resided closest to the border were more likely to be identified as imported malaria cases. Furthermore, proximity to larval habitats and forested areas can be a risk factor for locally transmitted cases. As Argentina obtained certification of malaria elimination in 2019, these results may inform focal strategies for preventing re-introduction, recognizing that insufficient malaria control on the Bolivian border represents a continuous risk. Therefore, sustained and strengthened cross-border cooperation is crucial not only to maintain malaria elimination status in Argentina but also to address the source of imported cases and advance towards regional elimination.

## Figures and Tables

**Table 1:** Total number of cases by demographic group, locality, and locality characteristic as well as the percentage of autochthonous and imported cases in each stratum near National Route 50, the Orán department, Northwestern Argentina, 1977–2009.

Variables		Total (% Analyzed by Column)	Autochthonous (% Analyzed by Row)	Imported (% Analyzed by Row)	*p*-Value
Individual-level Variables					
Sex	Female	483 (27.23%)	317 (65.63%)	166 (34.27%)	
	Male	1291 (72.77%)	947 (73.35%)	344 (26.50%)	<0.001
Nationality	Argentina	1620 (91.27%)	1213 (74.88%)	407 (25.12%)	
	Bolivia	144 (8.11%)	41 (28.47%)	103 (71.53%)	
	Paraguay	10 (0.56%)	10 (100%)	0 (0.00%)	<0.001
	Missing	1 (0.06%)	1 (100%)	0 (0.00%)	
Previous infection	No	1630 (91.83%)	1176 (72.15%)	454 (27.85%)	
	Yes	143 (8.06%)	88 (61.54%)	55 (38.46%)	0.022
	Do not know	2 (0.11%)	1 (50.00%)	1 (50.00%)	
Age groups	<5 years	154 (8.68%)	120 (77.92%)	34 (22.08%)	
	5–9 years	141 (7.94%)	104 (73.76%)	37 (26.24%)	
	10–19 years	325 (18.31%)	223 (68.62%)	102 (31.38%)	
	20–29 years	359 (20.23%)	250 (69.64%)	109 (30.36%)	0.021
	30–39 years	299 (16.85%)	196 (65.55%)	103 (34.45%)	
	≥40 years	497 (28.00%)	372 (74.85%)	125 (25.15%)	
Occupation	Homemaker	192 (10.82%)	138 (71.88%)	54 (28.13%)	
	Entrepreneur/merchant	75 (4.23%)	12 (16.00%)	63 (84.00%)	<0.001
	Domestic worker	66 (3.72%)	43 (65.15%)	23 (34.85%)	
	Day laborer	786 (44.28%)	602 (76.59%)	184 (23.41%)	
	Not working	101 (5.69%)	70 (69.31%)	31 (30.69%)	
	Other	125 (7.04%)	82 (0.6%)	43 (1.4%)	
	No occupation	430 (24.23%)	318 (73.95%)	112 (26.05%)	
Place of work	Argentina	1199 (67.55%)	962 (80.23%)	237 (19.77%)	
	Bolivia	497 (28.00%)	247 (49.70%)	250 (50.30%)	<0.001
	Missing	79 (4.45%)	56 (70.89%)	23 (29.11%)	
Origin of infection	Argentina	1028 (57.92%)	1018 (99.03%)	10 (0.97%)	
	Bolivia	452 (25.46%)	57 (12.61%)	395 (87.39%)	<0.001
	Missing	295 (16.62%)	190 (64.41%)	105 (35.59%)	
Home locality	Aguas Blancas	195 (10.99%)	123 (63.08%)	72 (36.92%)	
	El Cebilar	46 (2.59%)	35 (76.09%)	11 (23.91%)	<0.001
	El Pelícano	118 (6.65%)	96 (81.36%)	22 (18.64%)	
	Finca la Esperanza	21 (1.18%)	14 (66.67%)	7 (33.33%)	
	Hipólito Yrigoyen	52 (2.93%)	36 (69.23%)	16 (30.77%)	
	SRN Orán	825 (46.48%)	520 (63.03%)	305 (36.97%)	
	Peña Colorada	220 (12.39%)	182 (82.73%)	38 (17.27%)	
	Pichanal	38 (2.14%)	33 (86.84%)	5 (13.16%)	
	Río Blanco	109 (6.14%)	100 (91.74%)	9 (8.26%)	
	Río Pescado Puente	81 (4.56%)	71 (87.65%)	10 (12.35%)	
	Tabacal	70 (1.8%)	55 (78.57%)	15 (21.43%)	
Locality-varying Variables					
Distance from home to the forest	0: >5 km	1620 (91.27%)	1130 (69.75%)	490 (30.25%)	
	1: <5 km	155 (8.73%)	135 (87.10%)	20 (12.90%)	<0.001
Distance from home to larval habitats	0: >5 km	1927 (71.45%)	428 (22.21%)	1499 (77.79%)	
	1: <5 km	194 (15.55%)	82 (42.27%)	112 (57.73%)	0.696
Distance from home to crops	0: >5 km	1294 (72.90%)	882 (68.16%)	412 (31.84%)	
	1: <5 km	481 (27.10%)	383 (79.63%)	98 (20.37%)	<0.001
Distance from home to National Route 50	0: >1 km	972 (54.76%)	653 (67.18%)	319 (32.82%)	
	1: <1 km	803 (45.24%)	612 (76.21%)	191 (23.79%)	<0.001
Distance from home to the Bolivian border	0: >10 km	1175 (66.20%)	815 (69.36%)	360 (30.64%)	6.1663
	1: <10 km	600 (33.80%)	450 (75.00%)	150 (25.00%)	0.013

**Table 2:** Logistic regression analysis of place-level determinants of imported malaria cases near National Route 50, Orán department.

Variables	OR	95% CI
After 1996	5.46 ***	(4.34–6.87)
<5 km from forested area	0.19 ***	(0.09–0.38)
<5 km from larval habitats	0.19 ***	(0.07–0.54)
<5 km from crops	0.13 ***	(0.05–0.37)
<10 km from the border	1.55 *	(1.01–2.35)
<1 km from National Route 50	2.80 ^†^	(0.95–8.27)

OR: Odds ratio; CI: Confidence interval; ^†^ *p* < 0.10; * *p* < 0.05; *** *p* < 0.001.

**Table 3:** Multi-level models of change: analyzing the proportion of malaria cases imported across localities and time near National Route 50, Orán department, Northwestern Argentina, 1977–2009.

Fixed Effects—Initial Status	Model A	Model B	Model C
Intercept	0.3225 ** (0.0385)	0.0198 (0.0381)	0.0069 (0.0387)
1996 intervention			0.1883 (0.0466) ***
Male			−0.0149 (0.0521)
Place of work (ref = Argentina)			
Bolivia		0.0021 (0.0372)	
Missing		−0.0868 (0.0784)	
Origin of infection (ref = Argentina)			
Bolivia			0.4332 (0.0424) ***
Missing			0.1741 (0.0421) ***
Age groups (ref = <5)			
Age 5–9			0.1228 (0.0798)
Age 10–19			0.0289 (0.0587)
Age 20–29			−0.0178 (0.0585)
Age 30–39			−0.0122 (0.0591)
Age ≥ 40			0.0819 (0.0505)
Economic activity (ref = homemaker)			
Entrepreneur			0.0541 (0.1368)
Domestic worker			−0.1012 (0.0900)
Day laborer			−0.0385 (0.0710)
Not working			0.0147 (0.0817)
Other			−0.0031 (0.0829)
No occupation			−0.0201 (0.0546)
Rate of Change			
Intercept		0.0018 (0.0002) ***	0.0016 (0.0003) ***
Male			−0.0002 (0.0003)
Place of work (ref = Argentina)			
Bolivia Work			0.0003 (0.0002)
Missing Place of Work			0.0003 (0.0004)
Origin of infection (ref = Argentina)			
Bolivia Origin of Infection			−0.0012 (0.0002) ***
Missing Origin of Infection			−0.0008 (0.0002) ***
Age groups (ref = <5)			
Age 5–9			−0.0009 (−0.0004)
Age 10–19			−0.0002 (0.0003)
Age 20–29			−0.0003 (0.0003)
Age 30–39			−0.0005 (0.0003)
Age ≥ 40			−0.0006 (0.0003) *
Economic activity (ref = homemaker)			
Entrepreneur			0.0001 (0.0006)
Domestic worker			−0.0005 (0.0005)
Day laborer			0.0006 (0.0004)
Not working			−0.0002 (0.0004)
Other			0.0003 (0.0003)
No occupation			0.0001 (0.0003)
Variance Components			
Level 1—Within-locality	0.1940 *** (0.0110)	0.1587 ***(0.0090)	0.1038 *** (0.0060)
Level 2—In initial status	0.0117 ^~^ (0.0065)	0.0045 (0.0051)	0.0001 (0.0003)
Level 2—In rate of change		<0.0001(<0.0001)	<0.0001(<0.0001)
Covariance		<0.0001 (<0.0001)	<0.0001(<0.0001)
Pseudo R-squared			
Within-locality		0.1820	0.3459
Between-locality		0.6154	0.9778
AIC	785.4097	666.3496	461.2563

Model A is an unconditional means model; Model B is an unconditional growth model; and Model C contains a discontinuity term and demographic predictors. ^~^ *p* < 0.10; * *p* < 0.05; ** *p* < 0.01; *** *p* < 0.001.

## Data Availability

The datasets used during the current study are available from the corresponding author upon reasonable request.
